# Power and clinical utility of mesopic microperimetry analysis strategies in age‐related macular degeneration

**DOI:** 10.1111/aos.70008

**Published:** 2025-09-22

**Authors:** Francesco Cinque, Jeroen Pas, Mahfam Shahabi, Laurens Sluijterman, Sofie ten Brink, Anita de Breuk, Thomas J. Heesterbeek, Caroline Klaver, Carel Hoyng, Yara Lechanteur

**Affiliations:** ^1^ Department of Ophthalmology Donders Institute for Brain, Cognition and Behaviour, Radboud University Medical Center Nijmegen The Netherlands; ^2^ Section Biostatistics, IQ Health Radboud University Medical Center Nijmegen The Netherlands; ^3^ Department of Ophthalmology Erasmus Medical Center Rotterdam The Netherlands; ^4^ Department of Epidemiology Erasmus Medical Center Rotterdam The Netherlands; ^5^ Institute of Molecular and Clinical Ophthalmology Basel Switzerland

## Abstract

**Purpose:**

This study evaluates whether mesopic microperimetry (MMP) provides a more robust measure of retinal function compared to visual acuity (VA) in age‐related macular degeneration (AMD) clinical trials, with a focus on optimal analysis strategies.

**Method:**

Fellow‐eyes of unilateral neovascular AMD were prospectively studied. Presenting VA was measured. The Macular Integrity Assessment Microperimeter (MAIA) was used with a 4 to 2 staircase strategy with a 10° diameter grid containing 37 loci. Three analysis strategies were calculated: the mean of 37 sensitivity thresholds (MS), the per cent reduced threshold (PRT), and the log‐transformed candela mean (MS cd log). Sample size requirements were calculated for 12‐ and 24‐month follow‐ups using a paired one‐sided T‐test (*α* = 0.05, power = 0.80).

**Results:**

*N* = 123 were analysed (82 (65.5%) females; mean age 74.2 (7.8) years). Ranked high to low, the required sample size at 12 months was: MS (*n* = 51), MS cd log (*n* = 52), PRT (*n* = 139), and VA (*n* = 203). Similar trends were seen at 24 months, with MS requiring the smallest sample size (*n* = 85) and VA the largest (*n* = 1673).

**Conclusion:**

All MMP analysis strategies outperformed VA, and MS required the least number of patients to show significant changes. This trend was consistent for both 12 and 24 months. These findings provide strong statistical arguments for the use of MMP in longitudinal within‐subjects clinical trials and suggest that averaging decibels is optimal.

## 1 INTRODUCTION

Age‐related macular degeneration (AMD) is a progressive retinal disease and the major cause of irreversible visual impairment in the elderly in the Western world (Colijn et al., 2017). Presently, natural history studies and clinical trials evaluating potential new therapies are being conducted (Finger et al., 2019). To interpret outcomes of these studies, and to translate them to daily clinical practice, in‐depth understanding of the clinical endpoints is essential.

Current AMD trials use best‐corrected visual acuity (BCVA) as a functional endpoint (Csaky et al., 2017). However, BCVA has limitations as both a primary and secondary measure (Yang & Dunbar, 2021). It shows minimal change over typical trial durations due to its limited sensitivity to early and intermediate AMD, extra‐foveal geographic atrophy (GA), and even late AMD with foveal involvement (Pfau et al., 2021; von der Emde et al., 2019; Yang & Dunbar, 2021). BCVA cannot capture relevant extra‐foveal improvements in exudative AMD or adequately reflect slow foveal changes in GA, which often exceed trial durations (Lindner et al., 2017; Sleiman et al., 2017). Its inability to distinguish between AMD stages without foveal involvement may overlook critical pathology, hindering the evaluation of potential therapies (Sadda et al., 2016).

MMP—a relatively new functional retinal test—involves presenting light stimuli of varying intensity to predefined retinal locations, with the sensitivity threshold (candela/m^2^ expressed as decibels) algorithmically determined for each location (Pfau et al., 2021). MMP has been shown to be sensitive to progression while BCVA remained stable even in non‐advanced AMD (Hsu et al., 2019; Vujosevic et al., 2017; Wu et al., 2015). Based on these findings, MMP has been used as a secondary endpoint in clinical trials evaluating therapies for patients with GA secondary to AMD (Chang et al., 2024; Holz et al., 2018; Yang & Dunbar, 2021).

There has been growing interest in the optimal analysis strategy of MMP, venturing beyond either the mean of al sensitivity thresholds or a point‐wise cut‐off approach using absolute (0 db) or relative scotoma (e.g. <10 dB) (Chang et al., 2024; Pfau et al., 2021; Yang & Dunbar, 2021). The latter is criticized for obscuring true change, while the former might ‘miss’ localized pathology (Yang & Dunbar, 2021). While some proposed analysis strategy changes are specifically designed for GA, such as limiting the number of analysed sensitivity threshold values to those bordering GA, further general investigation is warranted for the use of MMP in other AMD stages (Chang et al., 2024; Csaky et al., 2019; Finger et al., 2019; Hsu et al., 2019).

The issue of random measurement error transcends disease stage and is relevant considering that the test–retest repeatability of the Macular Integrity Assessment microperimetry (MAIA; CentreVue, Padova, Italy) is approximately 4 dB and, in the case of deep scotoma including GA, 6 dB (95% coefficient of variation). These figures might be considered substantial relative to its 36 decibel scale (Pfau et al., 2021).

We hypothesize that random measurement error affects analysis strategies (mean or cut‐off) differentially. This will have consequences for statistical power and calculations of estimated annual change, both of which are essential for clinical trial design and interpretation of results. In this exploratory study, we explore three analysis strategies including the aforementioned mean, cut‐off, and a novel approach in a group of AMD patients from a prospective cohort study. The novel approach averages raw candela/m^2^ prior to log transformation. We hypothesize that the novel approach is more robust to random variation, resulting in a higher power to detect change over time while retaining sensitivity to local pathology.

## 2 METHOD

This secondary analysis was performed using patients with available follow‐up at 12 ± 2 months and 24 ± 2 months from the primary, ongoing, prospective cohort study with unilateral neovascular AMD patients. Details of the primary study are outlined below.

### 2.1 Primary study characteristics; design, setting, participants, and design

Participants were included between Jan 2018 and Nov 2022 and were evaluated every 6 months at the Radboud university outpatient clinic in Nijmegen. Participants left the study if the fellow‐eye became exudative and/or received anti‐vascular endothelial growth factor secondary to macular neovascularization. The study period encompassed 2 years after which participants were given the opportunity to continue or leave the study. Participants who left before 2 years without CNV in the fellow‐eye were asked to provide a reason. The aim of our study was to investigate AMD‐related changes prior to exudation in the fellow‐eye.

Patients with the following criteria were included: unilateral macular neovascularization (MNV) secondary to AMD without any ophthalmic complications in the fellow eye that would interfere with the study procedures, such as diabetic retinopathy, severe cataract, glaucoma and retinal vein occlusion. Potential participants were selected from referrals from ophthalmologists from neighbouring clinics, contact advertisements in patient association newsletters, and our own clinical population. This study was conducted in accordance with the tenets of the Declaration of Helsinki and was approved by the local ethics committee. All participants provided written informed consent prior to participating.

Medical and demographic information of each patient was collected using questionnaires. At each visit, visual acuity (VA) was measured using the Early Treatment Diabetic Retinopathy Study (ETDRS) chart. MMP was performed with the MAIA using a 4–2 staircase strategy with a grid of 10° diameter containing 37 radially oriented points centred on the fovea. A test run prior to the actual examination was performed. Participants were not in mydriasis (Han et al., 2017; Pfau et al., 2021). The grid was carefully aligned on the fovea. After mydriasis, multimodal imaging was performed, including colour fundus photography (CFP) (DRI Triton (Topcon corporation, Tokyo, Japan)) and spectral‐domain optical coherence tomography (SD‐OCT) (SpectralisTM HRA + OCT (Heidelberg Engineering, Heidelberg, Germany)).

### 2.2 MMP analysis strategies

**FIGURE 1:**
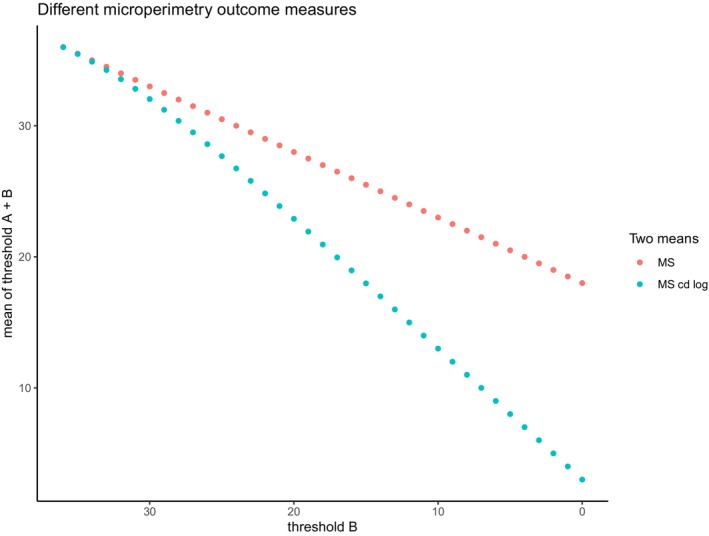
Demonstration of Difference between MS and MS cd log. In Figure 2 we demonstrate the difference between mean sensitivity or the mean of all 37 thresholds (MS) and MS cd log or the log‐transformed mean of candela values of 37 thresholds. Suppose we have a grid containing thresholds A and B. In our example, threshold A is fixated while threshold B decreases with a step of 1 dB. MS and MS cd log will behave differently as threshold B decreases. MS cd log will diverge from MS for lower dB values.

For the present analysis, three analysis strategies of MMP were calculated:

1. Mean sensitivity (MS): This is the average of all 37 sensitivity thresholds in dB.
2. Mean sensitivity candela log (MS cd log): MS cd log is calculated by first converting each sensitivity threshold into its corresponding candela/m^2^ value (Pfau et al., 2021). In step 2, all thresholds in candela/m^2^ are then averaged. The third step is to retransform the product of step 2 back to a dB value. The effect of this transformation relative to MS is outlined in Figure 1.
3. Percent reduced threshold (PRT): the number of sensitivity thresholds below 25 dB divided by 37 (the total number of thresholds), expressed as a decimal value.

### 2.3 Grading

CFP were graded by EYE‐NED grading centre (Rotterdam, the Netherlands) according to the Wisconsin age‐related maculopathy grading system (WARMGS) (Klein et al., 1991) and then converted to the Rotterdam study criteria (RS stage 0–4) (van Leeuwen et al., 2003).

### 2.4 Outcomes

The main outcome is the estimated sample size (power 80%) required to detect a statistically significant decline (at 0.05 level) in VA, MS, MS cd log, and PRT at 12 and 24 months. A relatively small sample size indicates that a decline in a certain analysis strategy is easier to detect than a decline in others. Similarly, in a fixed dataset, an analysis strategy with a smaller estimated sample size will have a lower chance of type II error.

Secondary outcomes were predicted annual change (95% CI) for each RS stage for VA, MS, MS cd log, and PRT using a mixed linear model.

### 2.5 Statistical methods

As a first, simple analysis, the average and variance of the paired differences at two different time points (12 and 24 months) were used to estimate the required sample size in VA, MS, MS cd log, and PRT. This was done for a power of 80% at a 0.05 significance level using a one‐sided t‐test.

Then, given the repeated measures design of the data, all timepoints were used in a mixed linear model. Estimated annual change of VA, MS, MS cd log, and PRT was modelled using a two‐level multilevel model as outlined by Singer and Willett (2003). We report our statistical output following the recommendations of Monsalves et al. (2020) Level 1 represents individual change of $Y_{\mathrm{ij}}$ in VA, MS, MS cd log, and PRT for time in years $t_{\mathrm{ij}}$. (1) Level 2 models the patient‐specific intercept $\beta_{0i}$ (2.1) and the patient‐specific slope $\beta_{1i}$ (2.2) for interindividual predictors RS stage 2 to 4 ($S_{i2}$, $S_{i3}$, $S_{i4}$) with RS stage 0 as reference. The full model is given by:

(1) $$Y_{\mathrm{ij}}=\beta_{0i}+\beta_{1i}t_{\mathrm{ij}}+\epsilon_{\mathrm{ij}},$$

and

(2.1) $$\beta_{0i}=\gamma_{00}+\gamma_{01}S_{i2}+\gamma_{02}S_{i3}+\gamma_{03}S_{i4}+u_{0i},$$

(2.2) $$\beta_{1i}=\gamma_{10}+\gamma_{11}S_{i2}+\gamma_{12}S_{i3}+\gamma_{13}S_{i4}+u_{1i}.$$

$\gamma_{00}$: average baseline value for patients with RS stage 0; $\gamma_{10}$: average slope for patients with RS stage 0; $\gamma_{01}$, $\gamma_{02}$, $\gamma_{03}$: differences in intercepts for RS stage 2,3, and 4 compared to RS stage 0; $\gamma_{11}$, $\gamma_{12}$, $\gamma_{13}$: changes for the slope for RS stage 2, 3, and 3 compared to RS stage 0.

The 95% CI and *p* values are reported. Goodness of fit was provided via marginal *R* ^2^ which assigns a percentage score to the variance focused of all fixed effects: time, RS stage, and time*RS stage; and secondly, conditional *R* ^2^ which represents the combined fixed and random effects (Nakagawa & Schielzeth, 2013). The variance partition coefficient was provided (variance between and within subjects) using a null model. All analyses were conducted in R 4.1.3 (2022‐03‐10) (R Core Team, Boston, MA, 2014). The lmer (1.1‐35.1), lmertest (3.1‐3), and the tidyverse packages (2.0.0) were used (Bates et al., 2015; Wickham et al., 2019).

## 3 RESULTS

### 3.1 Participants

During Jan 2018 and Nov 2022, 132 patients met the inclusion criteria for the primary study. Four patients developed bilateral exudative neovascular AMD between registration and first visit and were not invited for further participation. Also, MMP for 5 additional patients was not performed. The reasons were as follows: cognitive impairment/failure to grasp instructions (2), hemianopsia (1), bilateral leg amputation (1), unable to fixate (1). In total, *N* = 123 patients were selected for the present study. During follow‐up, 18 patients developed an MNV in the fellow eye. Twenty‐nine patients discontinued prematurely. The reasons were: death (7), travel distance (5), finding the study too burdensome (5), other non‐ophthalmic illnesses (4), participating in a rival ophthalmic study (2), other (6). Some patients failed to visit at the appointed intervals on account of covid restrictions but did not leave the study. Therefore, *n* = 68 patients were selected at 12 months and *n* = 31 at 24 months.

### 3.2 Descriptives

Of total (*N* = 123) mean (SD) age was 74.2 (7.8) years and 82 (67.5%) were female. The mean (SD) time since first eye involvement with neovascular AMD was 4.4 (4.1) years while the mean (SD) follow‐up time was 1.9 (1.2) years as of August 2023. At baseline, the fellow eye of 29 (23.6%) patients was graded as RS stage 0 corresponding to no signs of AMD at all or hard drusen (<63 μm) only, 0 (0%) as RS stage 1), 41 (33.3%) as RS stage 2, 32 (26.0%) as RS stage 3, and 21 (17.1%) as RS stage 4 or atrophic AMD. Additional demographic information can be found in Table 1.

**TABLE 1:** Baseline variables.

Variable	Value *N* = 123
Time since first eye involvement, mean (SD), years	4.4 (4.1)
Age baseline, mean (SD), years	74.2 (7.8)
Female, No. (%)	82 (67.5%)
Smoking, No. (%)	8 (6.5%)
Currently	76 (61%)
Ever	39 (33.5%)
Never	
University level education^a^	47/116 (38.2%)
Phakic, No. (%)	84 (68.9%)
RS stage 0–4, No. (%)	29 (23.6%)
RS stage 0	0 (0%)
RS stage 1	
RS stage 2	41 (33.3%)
RS stage 3	32 (26.0%)
RS stage 4	21 (17.1%)

Abbreviations: RS stage 0, corresponds to no signs of age‐related macular degeneration at all or hard drusen (<63 μm) only; RS stage 1, soft distinct drusen (≥63 μm) only or pigmentary irregularities only, no soft drusen (≥63 μm); RS stage 2, soft indistinct drusen (≥125 μm) or reticular drusen only, soft distinct drusen (≥63 μm); RS stage 3, soft indistinct (≥125 μm) or reticular drusen with pigmentary irregularities; RS stage 4, atrophic.

^a^ *n* = 7 missing values.

### 3.3 Change in VA and retinal sensitivity (MS, MS cd log, PRT)

**FIGURE 2:**
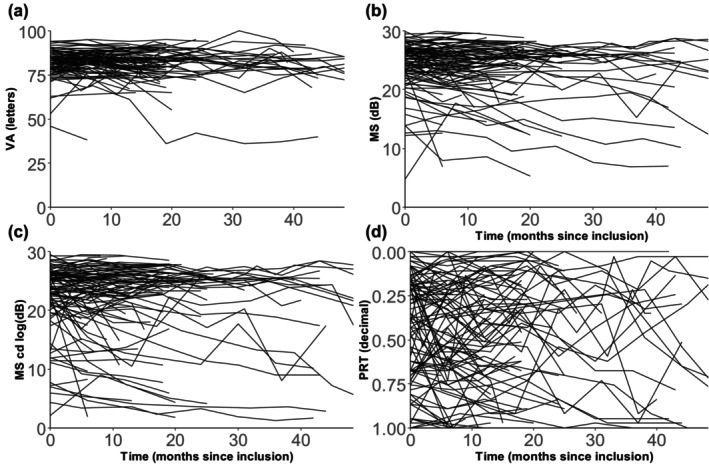
Longitudinal changes of VA, MS, MS cd log, and PRT. MS, mean sensitivity; MS cd log or mean sensitivity, candela log; PRT or per cent, reduced threshold; VA, visual acuity. Spaghetti plots showing longitudinal change for each analysis strategy.

At baseline mean (SD) VA was 81.9 (7.0) letters. Retinal sensitivity (mean (SD)) was 23.9 (4.9) dB, 22.5 (6.6) dB, and 0.4 (0.3) decimal value for MS, MS cd log, and PRT, respectively (Table 2). Mean loss (SD) of VA was 0.87 (5.0) and −0.45 (7.5) letters at 12 and 24 months. Retinal sensitivity change was −0.7 (2.0) dB for MS, −1.15 (3.3) dB for MS cd log, and 0.04 (0.2) decimal value for PRT at 12 months. At 24 months the change was −1.05 (3.9) dB, −1.10 (5.4) dB, and 0.04 (0.3) decimal value for MS, MS cd log, and PRT, respectively (Table 2). Longitudinal changes for VA, MS, MS cd log, and PRT are visualized in Figure 2 showing increasing instability for each subsequent analysis strategy. PRT clearly demonstrates the highest variability.

**TABLE 2:** Uncorrected changes and required sample size.

All stages	VA, letters mean (SD)	MS, dB mean (SD)	MS cd log, dB mean (SD)	PRT, dec. v. mean (SD)
Baseline	81.9 (7.0)	23.9 (4.9)	22.5 (6.6)	0.4 (0.3)
∆ 12 months	−0.87 (5.0)	−0.70 (2.0)	−1.15 (3.3)	0.04 (0.2)
∆ 24 months	−0.45 (7.4)	−1.05 (3.9)	−1.10 (5.4)	0.04 (0.3)
Required sample size, 12 months (*n* = 68)	203	51	52	139
Required sample size, 24 months (*n* = 31)	1673	85	152	318

*Note*: Required sample size indicates the number of patients based on 12 ± 2 months, 24 ± 2 months of available patients, we expect to need to include to have an 80% chance to detect a decline at 0.05 significance level using a one‐sided *t*‐test.

Abbreviations: dec. v., decimal value; MS, mean sensitivity; MS cd log, mean sensitivity candela log; PRT, per cent reduced threshold; VA, visual acuity.

### 3.4 Sample size

The estimated required sample size (Table 2) for VA is *n* = 203 at 12 months. This was *n* = 51 for MS, *n* = 52 for MS cd log, and *n* = 139 for PRT. At 24 months, *n* was 1673 for VA, *n* = 85 for MS, *n* = 152 for MS cd log, and *n* = 312 for PRT.

### 3.5 Estimated annual change

Intercepts for RS stage 4 were statistically different for MS (*p* < 0.001), MS cd log (*p* < 0.001), and PRT (*p* < 0.001) compared to all other stages. Similarly, intercepts for RS stage 3 were statistically different for MS (*p* = 0.01), MS cd log (*p* = 0.02), and PRT (*p* < 0.001) compared to all other stages (Table 3). Boxplots showing the distributions of VA and retinal sensitivity across RS stages are presented in Figure S1.

**TABLE 3:** Predicted changes of VA, MS, MS cd log and PRT per Stage.

Predictors	VA (letters)			MS (dB)			Ms cd log (dB)			PRT (decimal value)		
	Estimates	CI	*p*	Estimates	CI	*p*	Estimates	CI	*p*	Estimates	CI	*p*
Intercept	83.46			26.39			25.42			0.23		
Time (years)	0.9	−0.63 to 2.42	0.2	−0.26	−0.89 to 0.37	0.4	−0.29	−1.13 to 0.55	0.5	0.02	−0.02 to 0.06	0.4
RS stage 4	−3.53	−7.53 to 0.46	0.08	−6.83	−9.23 to −4.42	<0.001	−10.96	−14.00 to −7.91	<0.001	0.47	0.31 to 0.62	<0.001
RS stage 3	−2.63	−6.12 to 0.86	0.1	−2.72	−4.87 to −0.56	0.01	−3.24	−5.97 to −0.52	0.02	0.28	0.14 to 0.42	<0.001
RS stage 2	0.15	−3.20 to 3.49	0.9	−1.93	−3.96 to 0.10	0.06	−2.15	−4.73 to 0.42	0.1	0.13	0.00 to 0.26	0.06
Time × RS stage 4	−5.05	−7.51 to −2.58	<0.001	−1.11	−2.12 to −0.09	0.03	−1.07	−2.42 to 0.28	0.1	0.03	−0.03 to 0.10	0.3
Time × RS stage 3	−2.19	−4.40 to 0.02	0.05	−0.27	−1.16 to 0.62	0.6	−0.97	−2.16 to 0.22	0.1	0.02	−0.04 to 0.08	0.4
Time × RS stage 2	−1.82	−3.89 to	0.09	0.08	−0.72 to 0.92	0.9	0.08	−1.05 to 1.22	0.9	−0.01	−0.07 to 0.05	0.8
		0.25										
Marginal *R* ^2^/conditional *R* ^2^	0.180/0.782			0.352/0.889			0.394/0.884			0.274/0.821		

Abbreviations: MS, mean sensitivity; MS cd log, mean sensitivity candela log; PRT, percent reduced threshold; RS stage 0 corresponds to no signs of age‐related macular degeneration at all or hard drusen (<63 μm) only; RS stage 1, soft distinct drusen (≥63 μm) only or pigmentary irregularities only, no soft drusen (≥63 μm); RS stage 2, soft indistinct drusen (≥125 μm) or reticular drusen only, soft distinct drusen (≥63 μm); RS stage 3, soft indistinct (≥125 μm) or reticular drusen with pigmentary irregularities; RS stage 4, atrophic; VA, visual acuity.

In the MS model, annual change for RS stage 4 was estimated to be significantly higher compared to average change (−1.11 dB, 95% CI (−2.12 to −0.09), *p* < 0.03). No other MMP output variable showed a statistically significant interaction effect. Estimated annual change for RS stage‐4 was −1.07 dB 95% CI (−2.42 to 0.028) for MS cd log and in PRT this was 0.03 95% CI (−0.03 to 10) (Table 3). For VA, the estimated annual loss for RS stage 4 was 5.5 letters 95% CI (−7.53 to −2.58), *p* < 0.001).

The variance partition coefficient indicated that 69% of the variance for VA existed between patients, similarly these numbers were 80% for MS, 83% for MS cd log and 73% for PRT (Table S1). All models converged. Ranked highest to lowest, marginal *R* ^2^ was 0.394, 0.352, 0.272, 0.180 for MS cd log, MS, PRT and VA respectively.

## 4 DISCUSSION

In this secondary analysis of fellow eyes of 123 unilateral neovascular AMD patients, all MMP analysis strategies outperformed VA in terms of power (Hsu et al., 2019; Wu et al., 2015), and MS was most powerful (Table 2) This trend was consistent for both 12 and 24 months of follow‐up.

Estimated annual change provides additional insights into stage‐related change for each analysis strategy. All analysis strategies showed the fastest worsening for higher staged individuals over the course of the study as would be expected. However, only MS was statistically significant. For RS stage 4, estimated annual change was −1.11 dB 95% CI (−2.12 to – 0.09) for MS, while for MS cd log, this was −1.07 dB with a widened, insignificant 95% CI of −2.42 to 0.028. For PRT the 95% CI was even wider: −0.03 to 0.10 with an estimated annual change of 0.03.

Of all three analysis strategies, PRT was weakest in detecting longitudinal decline. Why is averaging optimal and why is PRT bad in terms of power? This is straightforwardly explained by the phenomenon that dichotomization of a continuous variable results in loss of statistical power (Altman & Royston, 2006). If the test value is erroneously measured below the true value and the cut‐off, this creates false positives and consequently inflates the number of ‘pathological’ points. If, however, the test value is in reality above the true value, this will result in false negatives. PRT, therefore, is uniquely susceptible to random error, thus weakening its ability to detect longitudinal decline.

Power issues likely persist when different cut‐off definitions are applied, such as age‐ and retinal locus‐specific reference thresholds (Charng et al., 2020). Therefore, pointwise evaluation, as Pfau et al. suggested, should only score values below the coefficient of variation as resulting from pathological change (Pfau et al., 2021). However, this approach would undermine the usefulness of MMP in detecting change, given that the average annual loss for intermediate AMD is −3.0 (3.3) dB (Hsu et al., 2019). Scoring only below the coefficient of variation may therefore obscure pathological changes. However, age‐specific PRT retains important advantages, particularly for between‐subject comparisons. It offers an intuitive, standardized method to identify functional deficits relative to normative populations and thus remains useful for regulatory and clinical decision‐making. The present critique is limited to its efficiency in detecting within‐subject longitudinal change, where MS may offer greater statistical power.

MS cd log can be viewed as a middle ground between MS and PRT. To our knowledge, this is the first application of the transformation in this context. The theoretical appeal lies in its tendency to produce lower values than MS in areas of poor sensitivity, while retaining similar values in regions of good sensitivity (see Figure 1). However, our findings do not show that MS cd log outperforms MS in terms of statistical power or stage differentiation. While we expected the transformation to improve robustness to random error and enhance sensitivity to scotoma formation, these benefits may be offset by increased variance in regions of low sensitivity. Nonetheless, it may be premature to dismiss this strategy, as it could prove valuable in alternative applications, such as detecting subtle changes in intermediate AMD.

### 4.1 Limitations

This study has several limitations. It relies on a non‐trial‐specific dataset and non‐AMD‐related factors, such as phakic changes, may influence MMP and VA measurements. However, the within‐dataset comparisons ensure generalizability. A minor limitation is the use of presenting visual acuity instead of BCVA, resulting in a suboptimal comparison between MMP and VA‐derived measurements. We acknowledge the use of different statistical approaches for power calculations (paired *t*‐tests) and secondary exploratory analyses (mixed‐effects linear models). While this could be seen as a methodological inconsistency, the power calculations were based on simple, conservative assumptions and are not the source of the large sample size estimates at later timepoints. Rather, these inflated estimates reflect the small number of available observations at 24 months (*N* = 31), a consequence of attrition and study design. Specifically, participants with longer follow‐up were more likely to have stable disease and no incident events such as MNV, introducing a selection effect that reduces longitudinal differences, thus inflating power requirements.

### 4.2 Future research

As part of MMP optimization, custom grids are increasingly employed and developed (Csaky et al., 2019). Following our results, custom grids would similarly benefit from averaging decibels, and an appropriate number of test loci would result in increasing robustness and power. Future efforts should ascertain an appropriate and optimal number of test loci, balancing test feasibility with statistical considerations. Importantly, methodological choices for a specific analysis strategy should be aligned with clinical relevance. This requires linking MMP change to patient‐reported outcomes in order to establish thresholds for what constitutes meaningful functional improvement or decline. While our findings support MS as the most efficient analysis strategy for detecting change, further research is needed to determine whether changes in MS reflect perceptible improvements in real‐world visual function (Evans, 2010; Fleming & Powers, 2012).

In sum, averaging threshold sensitivities results in the most stable outcome, detecting change with the least required sample size out of three analysis strategies. In addition, only MS was able to statistically discriminate higher staged longitudinal decline compared to other MMP derived analysis strategies. When detecting within‐subject change over time, trial designers should consider an averaging approach such as MS. However, age‐specific PRT may still be preferable for between‐subjects comparisons, especially against population norms. Secondarily, longitudinal trials should anticipate attrition and selection effects if events like MNV lead to study withdrawal.

## FUNDING INFORMATION

This research was supported by an Investigator Initiated Research sponsoring by Bayer. Bayer had no role in the design or conduct of this research. In addition, this work was supported by the following foundations: Gelderse Blindenstichting, Stichting Blindenhulp, Louise Rottinghuisfonds, and Landelijke Stichting voor Blinden en Slechtzienden, Algemene Nederlandse vereniging ter Voorkoming van Blindheid, Stichting Beheer Het Schild, and het Oogfonds that contributed through UitZicht. The funding organizations had no role in the design or conduct of this research. They provided unrestricted grants.

## Supporting information


Table S1.

Figure S1.

Figure S1 Legend.
