# Supplementary material for: Power and clinical utility of mesopic microperimetry analysis strategies in age‐related macular degeneration
Source: Acta Ophthalmol. 2025 Sep 22;104(3):e292–9. doi: 10.1111/aos.70008 (PMC13058685; doi:10.1111/aos.70008)
Supplement: Supplementary file 1 — Table S1. [file AOS-104-e292-s003.docx]

**Table s1. Null models for VA, MS, MS, cd log, and PRT**

|  | VA | MS | MS cd log | PRT |
| --- | --- | --- | --- | --- |
| Variance between patients | 44.8 | 17.3 | 37.7 | .08 |
| Variance within patients | 20.1 | 4.3 | 7.9 | .03 |
| VPC (between/between + within) | 69% | 80% | 83% | 73% |

VPC or variance partition coefficient for null models. VA visual acuity, MS or mean sensitivity, MS cd log or mean sensitivity candela log, PRT or percent reduced threshold.
