# Supplementary figures and images for: Power and clinical utility of mesopic microperimetry analysis strategies in age‐related macular degeneration
Source: Acta Ophthalmol. 2025 Sep 22;104(3):e292–9. doi: 10.1111/aos.70008 (PMC13058685; doi:10.1111/aos.70008)

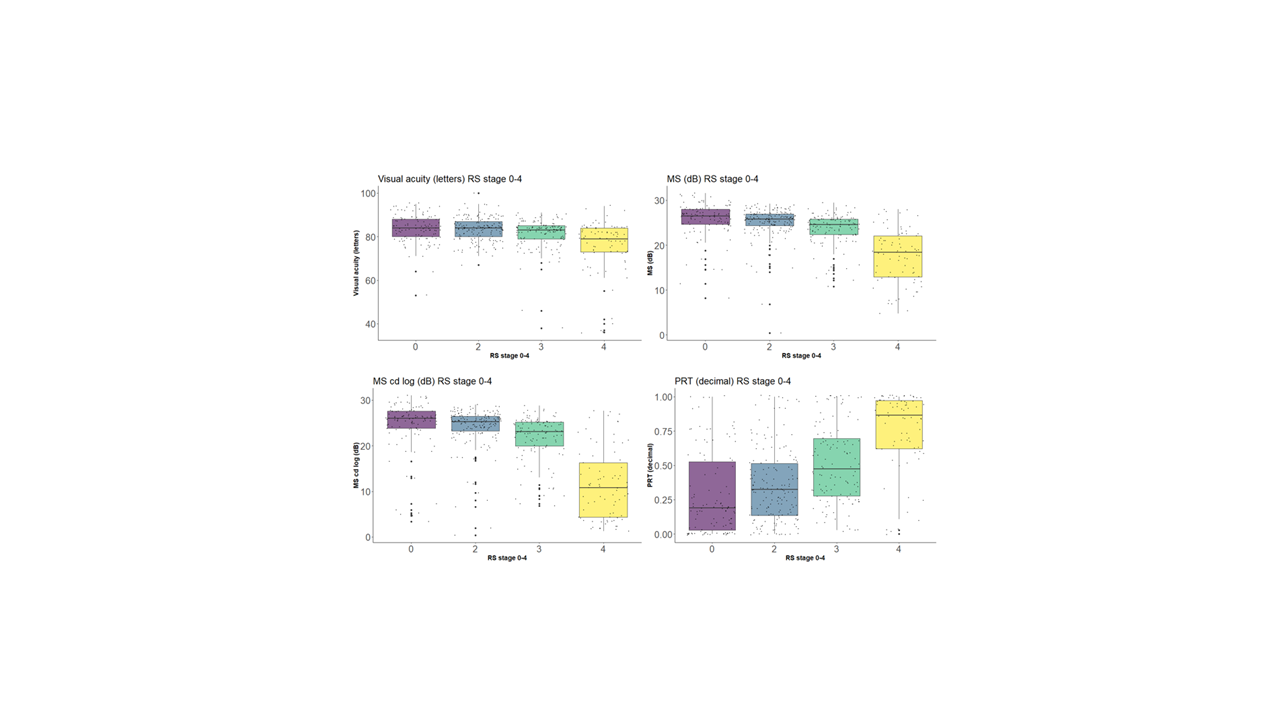

Supplement: Supplementary file 2 — Figure S1. [file AOS-104-e292-s002.tif]
