# Supplementary material for: Power and clinical utility of mesopic microperimetry analysis strategies in age‐related macular degeneration
Source: Acta Ophthalmol. 2025 Sep 22;104(3):e292–9. doi: 10.1111/aos.70008 (PMC13058685; doi:10.1111/aos.70008)
Supplement: Supplementary file 3 — Figure S1 Legend. [file AOS-104-e292-s001.docx]

**Figure s1. VA, MS, MS cd log, PRT at Baseline**

VA visual acuity, MS or mean sensitivity, MS cd log or mean sensitivity candela log, PRT or percent reduced threshold. In figure 4 baseline values for VA, MS, MS cd log, PRT, per stage (RS stage 0-4) are shown*.* RS stage 0 corresponds to no signs of AMD at all or hard drusen <63 µm) only. RS stage 1: Soft distinct drusen (≥63 µm) only or pigmentary irregularities only, no soft drusen (≥63 µm). RS stage 2: Soft indistinct drusen (≥125 µm) or reticular drusen only, soft distinct drusen (≥63 µm). RS stage 3: Soft indistinct (≥125 µm) or reticular drusen with pigmentary irregularities. RS stage 4: Atrophic or neovascular age-related macular degeneration.
